# Dietary Citrate Restores Age‐Related Endothelial Cell Mitochondrial Dysfunction and Alleviates Atherosclerosis

**DOI:** 10.1111/acel.70213

**Published:** 2025-09-04

**Authors:** Ya Zhao, Jia‐Yu Qiu, Fang Wu, Xue‐Ting Gong, Wei‐Xin Lv, Jian‐Kun Liu, Jia‐Yi Dong, Xue‐Er Li, An‐Dong Wu, Jing‐Jing Duan, Yang Xiang, Xiao‐Li Tian

**Affiliations:** ^1^ Aging and Vascular Diseases, Human Aging Research Institute (HARI) and School of Life Science Nanchang University, and Jiangxi Province Key Laboratory of Aging and Disease Nanchang Jiangxi China; ^2^ Human Aging Research Institute (HARI) and School of Life Science Nanchang University, and Jiangxi Key Laboratory of Aging and Disease Nanchang Jiangxi China; ^3^ Metabolic Control and Aging‐Jiangxi Key Laboratory of Aging and Diseases, Human Aging Research Institute (HARI), School of Life Science Nanchang University Nanchang China

**Keywords:** atherosclerosis, citrate, hypertension, mitochondrion, vascular aging

## Abstract

Vascular aging increases the susceptibility to cardio‐cerebrovascular conditions, such as atherosclerotic diseases and hypertension, the leading causes of global disability and mortality. Dietary citrate extends the lifespan of *Drosophila melanogaster* and *Caenorhabditis elegans* as well as improves the memory of mice injured by a high‐fat diet (HFD); whether it alleviates vascular aging and age‐related vascular diseases; however, remains unknown. Here, we showed that dietary supplementation of citrate delayed vascular aging, as evidenced by maintaining the integrity of elastic fibers and decreasing the level of the aging‐related marker, CDKN1A (p21). Functionally, citrate improved the sensitivity to endothelial‐dependent vasodilators and lowered blood pressure, and in HFD‐fed *ApoE* ^*−/−*^ mice, it reduced the size of atherosclerotic plaques, decreased the necrotic core area and vulnerability index in aortic root plaques. Additionally, citrate decreased the frailty index, increased bone density, and improved maximal grip strength and balance speed in both aged and HFD‐fed *ApoE* ^*−/−*^ mice. Mechanistically, we showed that citrate exposure delayed human umbilical vein endothelial cell senescence with a decreased percentage of cells stained with senescence‐associated β‐galactosidase and p21 levels. Moreover, citrate activated AMPK‐related pathways and reversed senescence‐related mitochondrial dysfunction in basal respiration, maximal respiration, and ATP production and reduced the production of reactive oxygen species (ROS). The citrate‐promoted beneficial effects were abolished due to inactivated AMPK and the increased mitochondrial ROS. Thus, we demonstrate that dietary citrate delays vascular aging and alleviates age‐related vascular diseases by improving mitochondrial function via activation of AMPK‐related pathways. Citrate may have potential clinical implications for interventions against vascular aging and age‐related vascular diseases.

## Graphical abstract

Citrate, as a common food additive, provides the major cellular ATP source. This study demonstrates that dietary citrate attenuates common vascular diseases, including atherosclerosis and hypertension, and reduces frailty in mice by delaying vascular aging. These beneficial effects are at least partially attributed to citrate‐mediated improvements in mitochondrial function and reduction in ROS, which are associated with the activation of AMPK signaling.

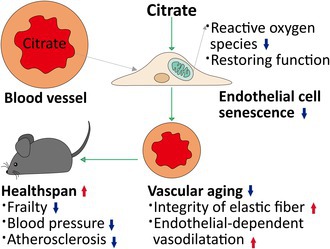

## 1 Introduction

Citrate, a common food additive, is one of the tricarboxylic acid cycle intermediates and provides the major cellular ATP source (Evans et al. 2010); it is mainly produced in the mitochondrion from acetyl‐CoA and oxaloacetate by citrate synthase (Iacobazzi and Infantino 2014). Previous studies reported that the level of citrate was decreased in human follicles with maternal aging, while citrate supplementation promoted oocyte maturation (He et al. 2022). Furthermore, dietary supplementation with citrate significantly extended lifespan in *Drosophila melanogaster* ( *D. melanogaster* ) (Fan et al. 2021), *Caenorhabditis elegans* ( *C. elegans* ) (Fei et al. 2004), and dose‐dependently improved metabolic health and memory in mice fed with a high‐fat diet (HFD) (Fan et al. 2021; Lin and Wang 2022).

Vascular aging is a well‐defined risk factor for common cardio‐cerebrovascular diseases, such as coronary artery disease, stroke, aneurysm, and hypertension (Laurent 2012; Long et al. 2022; Vanhoutte et al. 2017; Yu et al. 2024). An aged vessel presents several morphological and functional hallmarks (Tian and Li 2014). Morphologically, for instance, the disrupted elastic fibers and increased collagens, as well as thickened intima‐media layer and enlarged vascular lumen, are observed (Behnke et al. 2006; BonithonKopp et al. 1996; Jani and Rajkumar 2006). Functionally, the aged vessel becomes stiffer and presents contractile, particularly endothelium‐dependent contractile dysfunction (Heffernan et al. 2023; Reece and Hulse 2013; Vanhoutte 1988). Furthermore, an array of aging‐associated markers is elevated, such as cyclin‐dependent kinase inhibitor 1A (CDKN1A or p21) and 2A (CDKN2A or p16) (Mehdizadeh et al. 2022). These age‐related dysfunctions eventually increase the susceptibility to atherosclerosis or elevated blood pressure (Katsuumi et al. 2018; Long et al. 2022; Zhu et al. 2024). By far, it remains elusive whether dietary citrate is able to slow down vascular aging and age‐related vascular diseases; therefore, contributing to healthspan.

In this study, we decide to investigate the effects of citrate on vascular aging and age‐related vascular diseases in both naturally aged and HFD‐fed *ApoE* ^*−/−*^ mice and search for the possible mechanisms.

## 2 Results

### 2.1 Citrate Improves Physical Function and Reduces Frailty

**FIGURE 1:**
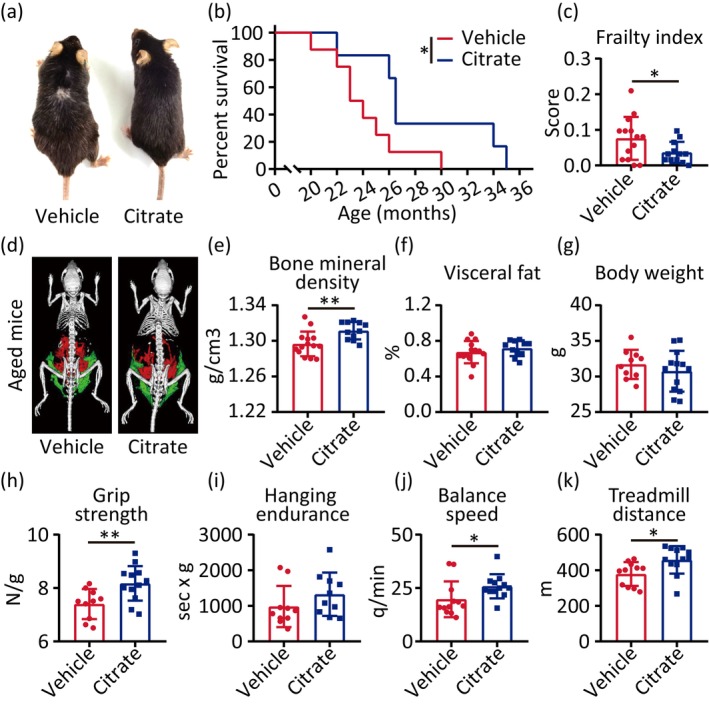
Citrate increases the functionalities and extends lifespan of aged mice. (a) Aged‐matched vehicle (left) and citrate‐fed (right) male mice, animals are 24 months old in the captured picture. (b) Survival curves of 18 months male mice treated with vehicle or citrate. (c–k) Frailty index (c), whole‐body micro‐CT images (d): bone (white), visceral fat (red), and subcutaneous fat (green), bone mineral density (e), visceral fat percentage (f), body weight (g), maximal grip strength (h), maximal hanging endurance (i), maximal balance speed (j), maximal treadmill distance (k) of 18 months male mice after 6‐month vehicle or citrate supplementation. Statistical analyses were executed using student's *t*‐test and log‐rank (Mantel‐Cox) test. Values are means ± SD. Vehicle: *n* = 8–14; Citrate: *n* = 6–13. *p < 0.05; **p < 0.01.

First, we determined the alterations of citrate in the serum of aged mice. Ultra‐high performance liquid chromatography–tandem mass spectrometry (UHPLC–MS/MS) was utilized for quantitative analysis of citrate in the serum of young (2 months) and aged (18 months) mice. The results showed that the serum citrate was significantly decreased in aged mice (Figure S1a). Then, we investigated the effects of citrate supplementation on lifespan; 1% citrate (w/v) was administered ad libitum in drinking water to male mice beginning at 18 months of age until natural death. Age‐matched control mice received water only. We found citrate supplementation significantly elevated serum citrate concentration in aged mice (Figure S1a), whereas it had no significant effect on food and water intake (Figure S2a–c). Finally, we examined the longitudinal effect of citrate supplementation on the healthy condition in the late lifespan of mice. Our data revealed that citrate supplementation significantly extended median and maximum lifespan in aged mice (Figure 1b). Citrate‐treated mice exhibited darker pelage pigmentation compared to the controls at age 24 months (Figure 1a) and a lower frailty index (Figure 1c). These findings suggest citrate attenuates age‐related physiological decline and extends healthspan in mice.

**FIGURE 2:**
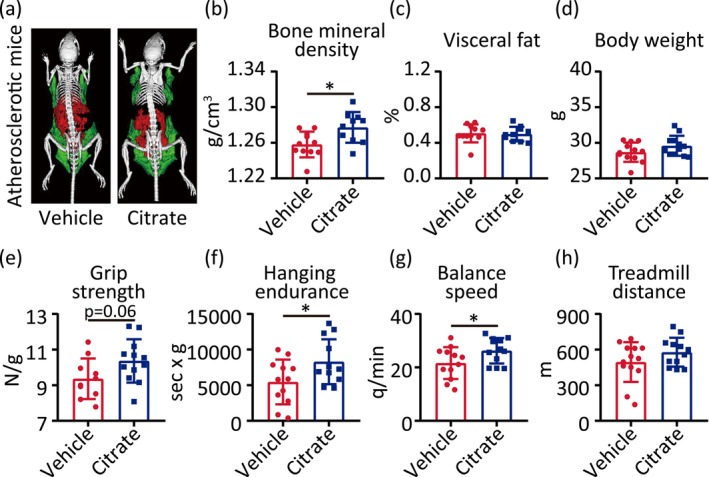
Citrate extends healthspan in atherosclerotic mice. (a) Whole‐body micro‐CT images from 8 weeks *ApoE* ^−/−^ male mice after 8‐week high fat diet and vehicle or citrate supplementation: bone (white), visceral fat (red) and subcutaneous fat (green). (b–h) Bone mineral density (b), visceral fat percentage (c), body weight (d), maximal grip strength (e), maximal hanging endurance (f), maximal balance speed (g), maximal treadmill distance (h) of 8 weeks *ApoE* ^−/−^ male mice after 8‐week high fat diet and vehicle or citrate supplementation. Statistical analyses were executed using student's *t*‐test. Values are means ± SD. Vehicle: *n* = 10–13; Citrate: *n* = 10–12. *p < 0.05.

Microcomputed tomography (micro‐CT) imaging of bone structure in naturally aged mice (24 months) showed that citrate treatment increased bone mineral density compared with vehicle (Figure 1d,e), but citrate had no effect on visceral fat distribution and body weight (Figure 1f,g). Functional assessments revealed increased maximal grip strength, maximal balance speed, and maximal treadmill distance in citrate‐treated aged mice relative to vehicle, but citrate had no effect on maximum hanging endurance (Figure 1h–k). Systemic evaluation of serum inflammation and lipid profiles revealed that citrate treatment significantly elevated high‐density lipoprotein cholesterol (HDL‐cholesterol) (Figure S3e), while exerting no discernible effect on high‐sensitivity C‐reactive protein (hs‐CRP), interleukin‐6 (IL‐6), tumor necrosis factor‐α (TNF‐α), matrix metalloproteinase‐9 (MMP‐9), low‐density lipoprotein cholesterol (LDL‐cholesterol), total cholesterol, and triglyceride (Figure S3a–d,f–h). These observations were validated in HFD‐fed *ApoE* ^−/−^ mice. We found that citrate treatment increased bone mineral density (Figure 2a,b), maximal grip strength, maximum hanging endurance, and maximal balance speed (Figure 2e–g), and decreased hs‐CRP (Figure S3i) compared with vehicle, but citrate had no effect on visceral fat distribution, body weight (Figure 2c,d), maximal treadmill distance (Figure 2h), IL‐6, TNF‐α, MMP‐9, HDL‐cholesterol, LDL‐cholesterol, total cholesterol, and triglyceride (Figure S3j–p).

These results suggest that citrate alleviates physical dysfunction and extends healthspan in both aged and atherosclerotic mice.

### 2.2 Citrate Alleviates Vascular Aging and Improves Endothelium‐Dependent Vascular Relaxation

**FIGURE 3:**
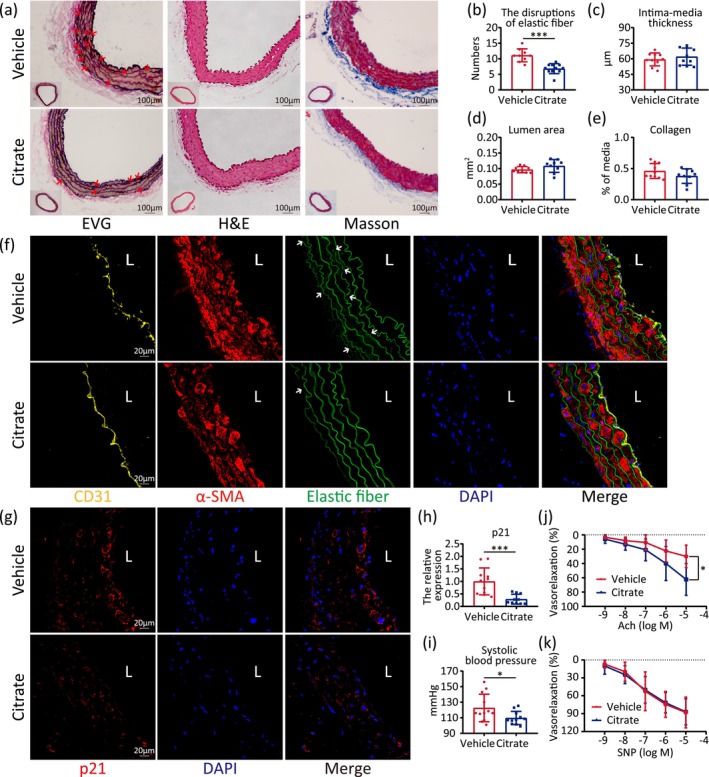
Citrate delays vascular aging and improves vascular function in aged mice. (a) Histological features of the aorta from 18 months male mice after 6‐month vehicle or citrate supplementation by staining of elastic van gieson (EVG) staining for elastic fibers (black), hematoxylin–eosin (H&E) staining for structure, and Masson's trichrome (Masson) staining for collagen (blue). (b–e) The number of disruptions per elastic fiber (b), intima‐media thickness (c), lumen area (d), and collagen proportion (e) of vehicle or citrate treatment. (f) Immunofluorescence staining of the aorta from vehicle or citrate‐fed mice by antibodies against CD31 (yellow) and α‐SMA (red). Elastic fibers (green) were labeled with spontaneous fluorescence, and nuclei (blue) stained by DAPI. (g, h) Immunofluorescence staining of the aorta from vehicle or citrate‐fed mice by antibodies against p21 (red). Nuclei (blue) stained by DAPI. Quantitative data for the relative expression of p21. (i) Systolic blood pressure of 18 months male mice after 6‐month vehicle or citrate supplementation. (j) Endothelium‐dependent vascular relaxation induced by acetylcholine (Ach) for the aorta rings of 18 months male mice after 6‐month vehicle or citrate supplementation. (k) Endothelium‐independent vasorelaxation mediated by nitroprusside (SNP) for the aorta rings of 18 months male mice after 6‐month vehicle or citrate supplementation. Statistical analyses were executed using student's *t*‐test and two‐way ANOVA. Values are means ± SD. Vehicle: *n* = 9–12; Citrate: *n* = 8–11. *p < 0.05; ***p < 0.001.

To evaluate whether citrate supplementation rescues aging‐related vascular functional decline, male mice at 18 months were supplied with 1% citrate in drinking water for 6 months. Morphological changes of aorta tissues were evaluated using elastic van Gieson (EVG), hematoxylin–eosin (H&E), and Masson's trichrome (Masson) staining (Figure 3a). Compared with non‐treated mice, the disrupted elastic fibers were reversed (Figure 3b) while intima‐media thickness, lumen area, and collagen content were not affected in the citrate‐treated group (Figure 3c–e).

We further demonstrated that citrate supplementation effectively ameliorated the disorganization of vascular smooth muscle cells (SMCs) and restored the integrity of the damaged intima layer (Figure 3f) and it reduced p21 expression and γ‐H2AX positive cell proportion significantly in aorta from aged mice (Figure 3g,h and Figure S4a,b).

To assess the potential of citrate to alleviate vascular dysfunction in aging mice, we conducted comprehensive evaluations of blood pressure and vascular contractile function. Our results indicated that citrate supplementation significantly reduced systolic blood pressure (Figure 3i) and markedly enhanced the vasodilatory response of aortic rings to acetylcholine (Ach) (Figure 3j), suggestive of the improvement of endothelium‐dependent vasorelaxation. However, the vasodilation of aortic rings in response to sodium nitroprusside (SNP) was comparable between control and citrate‐supplemented mice (Figure 3k), indicating that citrate specifically modulates endothelium‐dependent, rather than endothelium‐independent vasodilation.

### 2.3 Citrate Alleviates Atherosclerotic Plaque Formation and Enhances Plaque Stability

**FIGURE 4:**
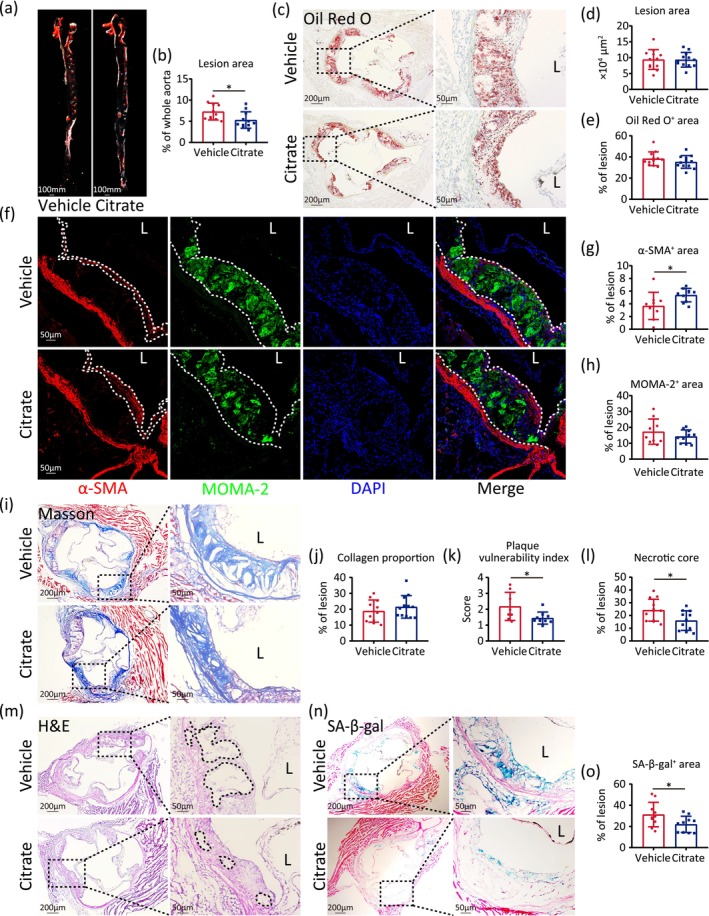
Citrate alleviates atherosclerosis and improves plaque stability. (a, b) Oil Red O staining of lesion area in the en‐face aortas from 8 weeks *ApoE* ^−/−^ male mice after 8‐week high fat diet and vehicle or citrate supplementation. Quantitative data for the percentage of lesion area in whole aorta. (c–e) Oil Red O staining of aortic root from vehicle or citrate treatment HFD‐fed *ApoE* ^−/−^ mice. Quantitative data for the lesion and Oil Red O positive area. (f–h) Immunofluorescence staining of aortic root from vehicle or citrate treatment HFD‐fed *ApoE* ^−/−^ mice by antibodies against α‐SMA (red) and MOMA‐2 (green). Nuclei (blue) stained by DAPI. Quantitative data for the percentage of α‐SMA and MOMA‐2 positive area lesion area in lesion area. (i–o) Masson's trichrome (Masson) staining for collagen proportion (i, j), quantitative data for vulnerable plaque index (k), hematoxylin–eosin (H&E) staining for necrotic core area (l, m), and SA‐β‐gal positive area (n, o) of aortic root from vehicle or citrate treatment HFD‐fed *ApoE* ^−/−^ mice. Statistical analyses were executed using student's *t*‐test. Values are means ± SD. Vehicle: *n* = 8–12; Citrate: *N* = 9–12. *p < 0.05.

To investigate the effects of citrate on atherosclerotic plaque formation, we administered 1% citrate‐supplemented drinking water or water alone to *ApoE* ^*−/−*^ mice fed with HFD. Analysis of aortic atherosclerotic lesions via Oil Red O staining revealed a significant reduction in the overall proportion of atherosclerotic lesions in the entire aorta of citrate‐treated HFD‐fed *ApoE* ^*−/−*^ mice compared to controls (Figure 4a,b). However, no significant differences were observed in lesion area or lipid content within the aortic root (Figure 4c–e). In addition, we demonstrated that citrate supplementation effectively restored the integrity of the damaged intima layer (Figure S5a). To further assess the impact of citrate on plaque stability, we evaluated plaque composition using immunofluorescence staining and histological analysis. The proportion of α‐smooth muscle actin (α‐SMA) and Ki‐67 positive SMCs was significantly increased within plaques of citrate‐treated mice relative to controls (Figure 4f,g and Figure S6a,b), indicating enhanced SMC content. In contrast, the proportion of macrophages, as detected by MOMA‐2 staining, did not differ significantly between groups (Figure 4f,h). Masson staining showed no significant changes in collagen content within the plaques (Figure 4i,j).

The aortic root of citrate‐treated HFD‐fed *ApoE* ^*−/−*^ mice had a lower vulnerability index than that of control (Figure 4k). Consistent with these findings, histological analysis of the entire lesion using H&E staining demonstrated a reduced proportion of necrotic core within plaques in citrate‐treated mice (Figure 4l,m). Additionally, the proportion of senescence‐associated β‐galactosidase (SA‐β‐gal)–positive cells was significantly decreased in citrate‐treated HFD‐fed *ApoE* ^*−/−*^ mice (Figure 4n,o).

### 2.4 Citrate Delays Vascular Endothelial Cell Senescence

**FIGURE 5:**
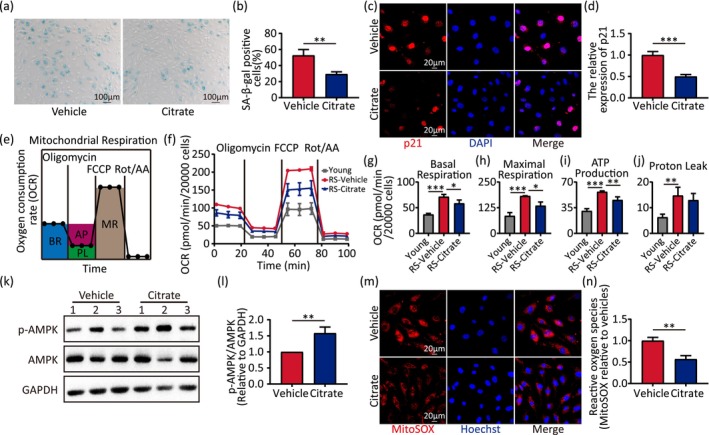
Citrate delays endothelial cell senescence and lessens senescence‐induced mitochondrial dysfunction. (a, b) SA‐β‐gal staining of senescent HUVECs treated with vehicle or citrate and quantitative data for SA‐β‐gal positive cells. (c, d) Immunofluorescence staining of senescent HUVECs treated with vehicle or citrate by antibodies against p21 (red), and nuclei (blue) stained by DAPI. Quantitative data for the relative expression of p21. (e) Schematic for the measurements of basal respiration (BR), ATP production (AP), proton leak (PL), and maximal respiration (MR). (f) Oxygen consumption rate (OCR) of young, replicative senescence (RS) and RS treated citrate in HUVECs. (g–j) Calculation of basal respiration (g), maximal respiration (h), ATP production (i), and proton leak (j) of young, RS and RS treated citrate in HUVECs. (k, l) Western blot analysis for the protein expression of p‐AMPK and AMPK in senescent HUVECs treated with vehicle or citrate. The p‐AMPK/AMPK ratio, normalized to GAPDH, were analyzed by densitometry, respectively. (m, n) ROS production of senescent HUVECs treated with vehicle or citrate by MitoSOX (red), and nuclei (blue) stained by Hoechst. Quantitative data for relative fluorescence of MitoSOX. Statistical analyses were executed using student's *t*‐test and one‐way ANOVA. Values are means ± SD. *p < 0.05; **p < 0.01; ***p < 0.001.

To determine the alterations of citrate in senescent cells, we cultured human umbilical vein endothelial cells (HUVECs) to a population doubling level (PDL) of 30 to establish a replicative senescence (RS) model and found citrate was significantly lower in senescent cells (PDL 30) compared to young cells (PDL 12) by UHPLC–MS/MS quantitative analysis (Figure S1b). To investigate whether citrate attenuates endothelial cell senescence, we first treated cells with serial concentrations of citrate (10 μM, 100 μM, 1 mM, 10 mM, and 100 mM). The results showed that exogenous citrate supplementation was able to restore the reduced citrate levels in senescent cells and identified 100 μM as the optimal concentration for cell proliferation analysis (Figures S1b and S7a,b). Next, we assessed cellular senescence markers, including SA‐β‐gal activity, p21 expression, and γ‐H2AX abundance. Exposure of HUVECs to citrate resulted in a significantly lower percentage of cells positive for SA‐β‐gal staining compared to controls (Figure 5a,b), and the decreased p21 protein (Figure 5c,d) and γ‐H2AX level (Figure S4c,d). Our data suggest that citrate delays the onset of endothelial cell senescence.

### 2.5 Citrate Reverses Senescence‐Related Mitochondrial Dysfunction

To evaluate the impact of citrate on mitochondrial bioenergetics, we quantified oxygen consumption rates (OCR) using the Seahorse XF Analyzer in young (PDL 12), untreated RS (PDL 30), and citrate‐treated RS endothelial cells, and OCR values were normalized to cell number to derive parameters including basal respiration, maximal respiration, ATP production, and proton leak (Figure 5e,f).

RS cells exhibited elevated basal respiration (Figure 5g), maximal respiration (Figure 5h), ATP production (Figure 5i), and proton leak (Figure 5j) compared to young controls. Citrate supplementation significantly attenuated these senescence‐associated hypermetabolic phenotypes, reducing basal respiration, maximal respiration, and ATP production.

To probe mechanistic links, we evaluated AMPK activation and mitochondrial reactive oxygen species (ROS) dynamics. Western blot analysis revealed a significant increase in the p‐AMPK/AMPK ratio in citrate‐treated RS cells versus vehicle controls (Figure 5k,l), indicating AMPK pathway activation. Concurrently, MitoSOX Red fluorescence (a mitochondrial superoxide‐specific probe) demonstrated a remarkable reduction in ROS levels with citrate treatment (Figure 5m,n).

### 2.6 Reducing ROS Is Critical to Citrate‐Delayed Senescence

**FIGURE 6:**
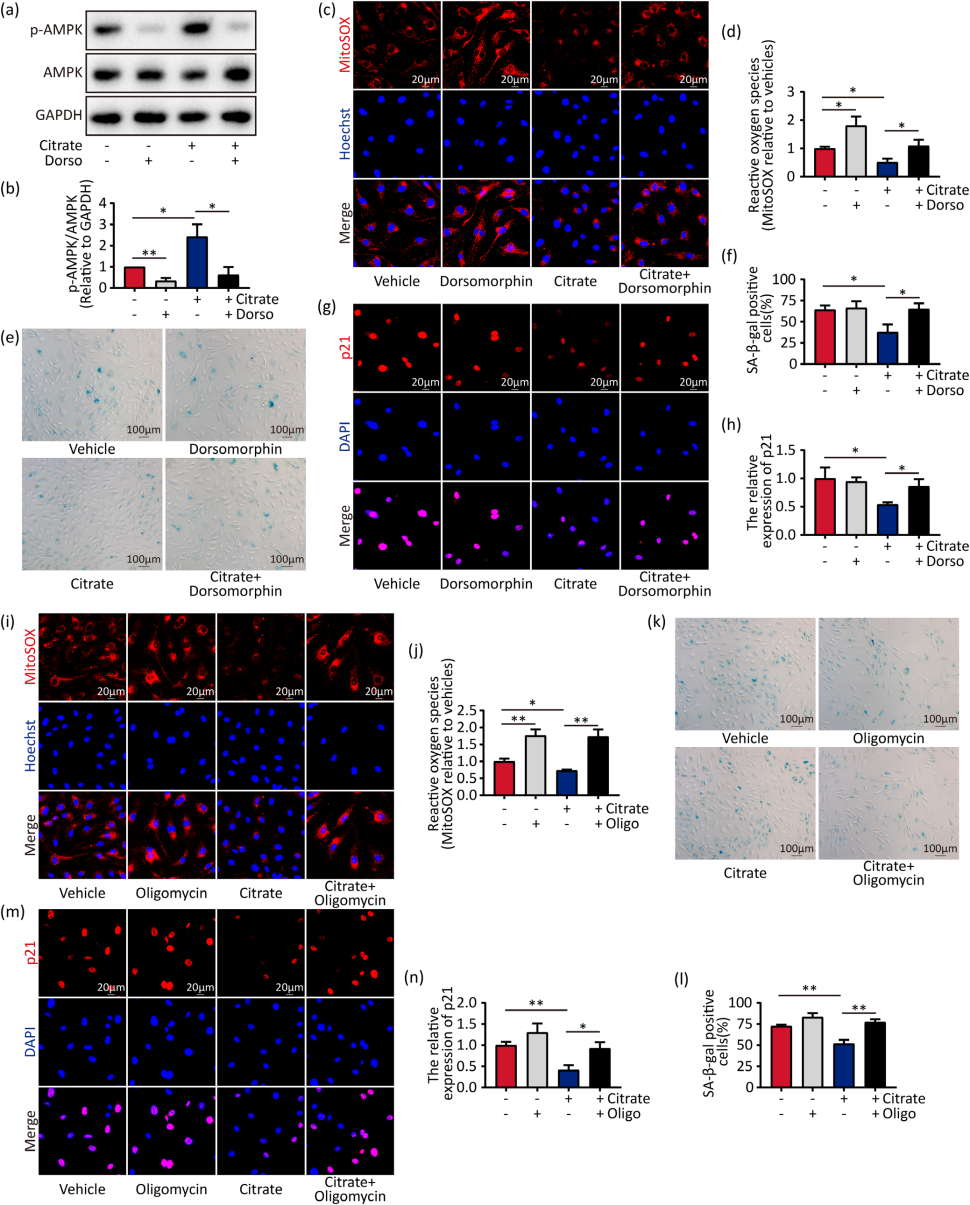
Impact of AMPK‐ROS on the development of citrate‐delayed senescence. (a, b) Western blot analysis for the protein expression of p‐AMPK and AMPK in senescent HUVECs treated with citrate or/and dorsomorphin. The p‐AMPK/AMPK ratio, normalized to GAPDH, were analyzed by densitometry, respectively. (c, d) ROS production of senescent HUVECs treated with citrate or/and dorsomorphin by MitoSOX (red), and nuclei (blue) stained by Hoechst. Quantitative data for relative fluorescence of MitoSOX. (e, f) SA‐β‐gal staining of senescent HUVECs treated with citrate or/and dorsomorphin and quantitative data for SA‐β‐gal positive cells. (g, h) Immunofluorescence staining of senescent HUVECs treated with citrate or/and dorsomorphin by antibodies against p21 (red), and nuclei (blue) stained by DAPI. (i, j) ROS production of senescent HUVECs treated with citrate or/and oligomycin by MitoSOX (red), and nuclei (blue) stained by Hoechst. Quantitative data for relative fluorescence of MitoSOX. (k, l) SA‐β‐gal staining of senescent HUVECs treated with citrate or/and oligomycin and quantitative data for SA‐β‐gal positive cells. (m, n) Immunofluorescence staining of senescent HUVECs treated with citrate or/and oligomycin by antibodies against p21 (red), and nuclei (blue) stained by DAPI. Statistical analyses were executed using one‐way ANOVA. Values are means ± SD. *p < 0.05; **p < 0.01.

To investigate whether citrate‐delayed senescence is dependent on AMPK, HUVECs were treated with dorsomorphin, an inhibitor of AMPK, alone or in combination with citrate. It was found dorsomorphin successfully reduced the p‐AMPK/AMPK ratio induced by citrate (Figure 6a,b) and it significantly increased mitochondrial ROS level (Figure 6c,d), SA‐β‐gal positive cells (Figure 6e,f), and p21 protein (Figure 6g,h) in citrate‐treated HUVECs.

We tested if mitochondrial ROS mediates the signaling of citrate‐delayed senescence; we treated HUVECs with oligomycin (to increase ROS) and/or citrate and found that mitochondrial ROS level was elevated (Figure 6i,j) and that SA‐β‐gal positive cells (Figure 6k,l) and p21 protein (Figure 6m,n) were significantly increased in the group treated with both oligomycin and citrate compared to that in the citrate group.

Thus, our results support that citrate delays endothelial cell senescence through the AMPK‐ROS signaling cascade.

## 3 Discussion

In this study, we demonstrate that dietary citrate attenuates common vascular diseases, including atherosclerosis and hypertension, and reduces frailty in mice by delaying vascular aging. These beneficial effects are at least partially attributed to citrate‐mediated improvements in mitochondrial function and reduction in ROS, which are associated with the activation of AMPK signaling. These findings suggest that citrate holds potential for the prevention of age‐associated vascular dysfunction and frailty.

Citrate, synthesized in the mitochondria from acetyl‐CoA and oxaloacetate via citrate synthase (Iacobazzi and Infantino 2014), serves dual roles as a major precursor for ATP production and as an indicator of cellular energy status (Evans et al. 2010). In the cytosol, citrate is metabolized to acetyl‐CoA, which participates in various biological processes, including sterol and fatty acid synthesis, as well as protein acetylation (Majd et al. 2018).

The cytosolic citrate level is regulated through multiple processes. For example, the mitochondrial citrate carrier (SLC25A1) transports citrate from the mitochondria to the cytosol (Ohanele et al. 2024), cellular membrane citrate transporters, such as SLC13A5 and ANKH/SLC62A1, mediate citrate influx and efflux, respectively (Birkenfeld et al. 2011; Szeri et al. 2020), and cytosolic ATP‐citrate lyase (ACLY) catalyzes citrate to acetyl‐CoA and oxaloacetate (He et al. 2022).

Disturbance of cytosolic citrate levels by genetic or pharmacological modification of these transporters and enzymes has profound pathophysiological consequences. It was reported that genetic variants of ACLY, which mimic the effects of ACLY inhibitors, decreased LDL cholesterol levels and reduced cardiovascular disease risk in a large population‐based study (Ference et al. 2019). In granulosa cells, age‐related increases in ACLY activity decrease citrate levels, impairing oocyte quality, while enhanced autophagy‐mediated reduction of ACLY protein restores oocyte quality (He et al. 2022). Conversely, ACLY knockdown promotes senescence in various cancer cell lines (Wei et al. 2021). Additionally, knockout of the SLC62A1 (ANKH) gene, which prevents citrate efflux from cells, has been reported to promote aortic aneurysm formation (Wu et al. 2024). Actually, inhibition of either ACLY or ANKH is supposed to increase cytosolic citrate levels. However, the divergent outcomes observed in these studies highlight the complexity of citrate metabolism and its downstream effects.

Interestingly, the loss‐of‐function of *SLC13A5* or orthologous genes, which prevent the cytosolic influx of citrate and are supposed to decrease cytosolic citrate, has a consistent beneficial effect on lifespan and metabolic health similar to caloric restriction across species in model organisms, including mice (*Slc13a5*), *D. melanogaster* (*Indy*) and *C. elegans* (*nac‐2*) (Birkenfeld et al. 2011; Rogina et al. 2000; Schwarz et al. 2015).

Dietary citrate supplementation has been demonstrated to be beneficial in various studies. For example, it enhances longevity as well as metabolic health in both fly and mouse model (Fan et al. 2021). The activation of citrate synthase by the dietary citrus flavanone naringenin prolongs the lifespan and healthspan of *C. elegans* and improves brain function in aged mice (Piragine et al. 2024). Another study demonstrated that HydroZitLa, a beverage enriched with citric acid, delays cellular senescence in human kidney cells (HK‐2) and extends the median lifespan of *C. elegans* (Lordumrongkiat et al. 2022). We in this study showed that dietary citrate delays vascular aging. This conclusion is based on the following observations: first, citrate maintains the integrity of vascular elastic fibers, which is typically impaired with age, and inhibits the expression of the aging‐related marker CDKN1A. Then, vascular endothelium‐dependent vasodilation, which becomes desensitized with age, is restored by citrate. Finally, atherosclerosis and hypertension, two age‐related vascular diseases, are significantly alleviated in both natural aging and HFD‐fed mouse models deficient in the *Apoe* gene. In addition, citrate supplementation decreases the frailty index, increases bone density, and improves maximal grip strength and balance speed in both models. Although dietary citrate is supposed to exert an overall effect at the individual level, it has been noted that dietary citrate‐induced beneficial effects are similar to those observed under dietary restriction conditions (Lin and Wang 2022). The mechanism, however, remains unclear.

Cytosolic citrate directly regulates cellular metabolism by inhibition of glycolysis and induction of gluconeogenesis (Icard et al. 2023). Indirectly, citrate can be converted into acetyl‐CoA, critical to the acetylation‐mediated regulations (Wellen et al. 2009; Wu et al. 2024). The effect of citrate likely depends on cellular energy status and adaptive responses. For example, tumor cells or tissues frequently have an increased expression of ACLY, while the knockdown of ACLY, supposed to increase the cytosolic citrate, inhibited PI3K–AKT but activated AMPK‐ROS pathway, promoting cancel cell senescence (Wei et al. 2021). Differently, the age‐increased ACLY decreases citrate level and impair oocyte quality during human aging (He et al. 2022). Interestingly, the deficiency in SLC13A5 orthologous genes, supposed to decrease the cytosolic citrate, activates AMPK pathways across species to extend lifespan (Schwarz et al. 2015). Mechanistically similar to the condition of “decreased cytosolic citrate,” that is, the dietary citrate activated AMPK (increased phosphorylated AMPK), suppressed target of rapamycin (TOR) signaling, and enhanced ketogenetic pathways in *D. melanogaster* (Fan et al. 2021). Here, we presented that citrate delayed endothelial cell senescence and vascular aging through alleviating age‐related mitochondrial dysfunction as it normalized respiratory hyperactivation, enhanced AMPK signaling, and suppressed mitochondrial ROS overproduction, which were associated with an increased phosphorylated AMPK. Citrate delays vascular aging and lowers blood pressure, contributing to the reduced atherosclerotic plaque size and enhanced plaque stability.

These observations also suggest that citrate‐associated effects may exhibit variability contingent upon the cell type, tissue, or even species. This is because energy expenditure and production diverge across these different contexts. Consequently, prudence must be exerted when elucidating the function of citrate (Ashbrook et al. 2015; Hong et al. 2023; Leandro et al. 2016; Mishra et al. 2021; Mycielska et al. 2022; Zhao et al. 2021).

While our study is the first to demonstrate that dietary citrate delays vascular aging and its associated consequences, human population‐based trials are still needed for validation. On the other hand, we should be aware that the systemic beneficial effects of dietary citrate may not be solely attributed to improved vascular conditions. Instead, they likely represent the cumulative impact of multiple organs and tissues. This is because citrate serves as a common energy sensor and regulator. Furthermore, the current study was focused on elastic vessels; how citrate affects muscular vessels or capillaries requires further investigation. In addition, although we showed that citrate significantly influences mitochondrial function, including ROS production, we did not perform tracer studies on citrate due to technical difficulties. Therefore, it remains unclear whether citrate acts directly on mitochondria or indirectly via cellular mechanisms.

In summary, our findings indicate that citrate alleviates age‐associated vascular dysfunctions by protecting mitochondria. These results suggest potential clinical applications of citrate supplementation for interventions against vascular aging and age‐related vascular diseases.

## 4 Experimental Procedures

### 4.1 Animal Care and Use

All animal experiments were approved by the Institutional Animal Care and Use Committee of Nanchang University (Approval No. NCULAE‐20221130017). Animals were acclimated for at least 1 week in a specific pathogen‐free animal facility maintained under a 12‐h light/dark cycle at 22°C–24°C. In addition, animals were group‐housed (1–4 mice per cage) in standard polycarbonate cages with sterile bedding and were provided with autoclaved food and fresh water.

Aged male C57BL/6 mice were purchased from Nanjing Junke Bioengineering Corporation Ltd. (Nanjing, China) fed with fresh water and food available until 18 months. Then they were randomly assigned to the vehicle group (fresh water, replaced daily) and the citrate group (water with 1% sodium citrate, replaced daily). Both groups were treated for a duration of 6 months or until natural death.

*ApoE* ^−/−^ male C57BL/6 mice were purchased from Gempharmatech Corporation Ltd. (Nanjing, China) fed with fresh water and food available until 8 weeks. Then they were randomly assigned to vehicle group (fresh water, replaced daily) and citrate group (water with 1% sodium citrate, replaced daily). Both groups began high fat diet and continued for 8 weeks to establish aortic lesions.

### 4.2 Serum Sample

Blood was collected from mouse orbital sinuses using a needle, and samples were allowed to clot at room temperature for 30 min. Clotted blood was centrifuged at 3000 rpm for 7 min at 25°C, and the supernatant was transferred to a new tube. This centrifugation step was repeated twice. Serum was mixed with ice‐cold methanol (1:4 v/v) for protein precipitation, incubated on ice for 5 min, and centrifuged at 20,000*g* for 20 min at 4°C. The supernatant was transferred to a vacuum concentrator and dried under reduced pressure at 25°C. Dried pellets were reconstituted in deionized water.

### 4.3 Cytosolic Sample

Briefly, cell lysis buffer was added to a 6‐cm culture dish, and cells were incubated on ice for 30 min. The lysate was centrifuged at 800 g for 10 min at 4°C to separate the supernatant, followed by a second centrifugation at 10,000*g* for 20 min at 4°C to separate the supernatant. The supernatant was mixed with ice‐cold methanol (1:4 v/v) for protein precipitation, incubated on ice for 5 min, and centrifuged at 20,000*g* for 20 min at 4°C. The supernatant was transferred to a vacuum concentrator and dried under reduced pressure at 25°C. Dried pellets were reconstituted in deionized water.

### 4.4 Ultra‐High Performance Liquid Chromatography–Tandem Mass Spectrometry (UHPLC–MS/MS)

Citrate concentrations were determined by UHPLC–MS/MS on a ShimadzuUHPLC‐40 LC system (Shimadzu corporation) and a Triple TOF5500+ (AB company) mass spectrometer. The chromatographic conditions were optimized, and finally, separation was performed on an ACQUITY UPLC HSS T3 column (100 mm × 2.1 mm, 1.8 μm) at 30°C with a mobile phase flow rate of 0.2 mL/min. The mobile phase consisted of 20 mM ammonium acetate‐H_2_O (mobile phase A) and acetonitrile (mobile phase B). A linear gradient elution program was adopted as follows: 0–2 min, 0% B; 2–2.5 min, 0%–90% B; 2.5–5 min, 90% B; 5–5.1, 90%–0% B; 5.1–8 min, 0% B. The injection volume was 5.0 μL. Citrate was detected in negative ionization mode. The parameters were set as follows: IsoSpray Voltage, −4500 V; Temperature, 500°C; Ino Source Gas 1, 50 psi; Ino Source Gas 2, 50 psi; Curtain GAS, 30 psi; Parent ion, 191.0 m/z; Quantitative ion, 111.2 m/z; Qualitative ion, 172.6 m/z; Collision energy, −18 eV; Declustering potential, −36 V. Peak integration and quantification of citrate were performed using SCIEX OS software.

### 4.5 Quantification of Inflammation and Plasma Lipid Fractions

Quantification of hs‐CRP, IL‐6, TNF‐α, MMP‐9, HDL‐cholesterol, LDL‐cholesterol, total cholesterol, and triglyceride in plasma from mice was performed using commercially available kits (Meimian, MM‐0372M2, MM‐1001M2, MM‐0132M2, MM‐20918R2; Nanjing jiancheng, A112‐1‐1, A113‐1‐1, A111‐1‐1, A110‐1‐1).

### 4.6 Frailty Index

The frailty index assessment and scoring followed a previously described protocol (Whitehead et al. 2014). To ensure objectivity in test interpretation, all phenotypic measurements were performed in a fully blinded manner. These assessments indicated age‐related deterioration of health and included evaluation of the animal musculoskeletal system, the vestibulocochlear/auditory systems, the ocular and nasal systems, the digestive system, the urogenital system, the respiratory system, signs of discomfort, body weight, and body surface temperature. The severity of frailty score constituted a simple 0–1 score per physical composition item, in which 0 indicated no sign of deficit, 0.5 indicated mild deficit, and 1 indicated severe deficit.

### 4.7 Micro‐Computed Tomography (Micro‐CT) Measurements

Micro‐CT scanning was performed using the Quantum GX3 microCT system (PerkinElmer). Briefly, anesthetized mice were placed inside the imaging machine in a prone position. Data obtained included bone mineral density (BMD) and fat mass.

### 4.8 Grip Strength Measurement

Grip strength measurement was performed utilizing a grip dynamometer (Karwin). Prior to testing, mice were habituated to the procedure for 3 days. Mice were tested in five trials, and the mean value of the maximum grip strength was calculated.

### 4.9 Hanging Endurance Measurement

Hanging endurance measurement was performed utilizing the barbed wire. Prior to testing, mice were habituated to the procedure for 3 days. Mice were tested for three trials, and the mean value of the maximum hanging endurance was calculated.

### 4.10 Rotated Test

Rotated test was performed utilizing a KW‐6C model (Karwin). Prior to testing, mice were habituated to the procedure for 3 days. The mice were placed on the rotating rod with an initial rotational speed of 6 rpm, and the acceleration rate was set at 2 rpm per minute. The test persisted until the mouse fell off the rod, and the maximum balance speed was recorded.

### 4.11 Treadmill Test

Treadmill test was performed utilizing a KW‐PT machine (Karwin). Prior to testing, mice were habituated to the procedure for 3 days. The mice were placed on the treadmill with an initial speed of 5 m/min, and the acceleration rate was set at 1 m/min per minute. The test persisted until the mouse ceased running, and the maximum treadmill distance was recorded.

### 4.12 Histochemistry

The thoracic aortas from aged mice were fixed in 4% paraformaldehyde (PFA) for 24 h and then embedded in paraffin. The aorta sections were 7 μm thick and stained with EVG staining for elastic fibers, H&E staining for structure, and Masson staining for collagen deposition. Images were acquired with a microscope and analyzed by ImageJ.

The aortic root and the entire aorta from HFD‐fed *ApoE* ^−/−^ mice were fixed with 4% PFA for 24 h, and the frozen sections were prepared with the aortic root in 7 μm thickness. Frozen sections of the aortic root and the entire aorta were subsequently stained with Oil red O for quantification of the lesion area, H&E staining for the necrotic core, and Masson staining for collagen deposition. Images were taken under a light microscope. The lesion area and proportion of each plaque component were analyzed by ImageJ. Plaque stability was evaluated by comparing the percentages of the above plaque components in the entire plaques. The histological plaque vulnerability index was calculated as follows: plaque vulnerability index = (macrophage proportion + lipid proportion)/(α‐SMA proportion + collagen proportion).

### 4.13 Blood Pressure Measurement

Systolic blood pressure was measured in mice using a non‐invasive tail‐cuff device IITC (Life science). Prior to experimental recordings, animals underwent 1‐week acclimatization protocol involving daily exposure to the restraint apparatus and cuff inflation procedures without data acquisition. Quantitative measurements were subsequently conducted daily over three consecutive days. Each measurement cycle consisted of a 10–15 s inflation‐deflation interval, with data acquisition parameters set to capture 10 consecutive pulsatile waveforms per experimental subject. All hemodynamic recordings were performed under standardized environmental conditions with continuous monitoring.

### 4.14 Isometric Tension Measurement

Thoracic aortas from mice were harvested and perivascular adipose tissues were meticulously removed in modified Krebs–Henseleit solution (stock = 118 mM NaCl/4.7 mM KCl/2.5 mM CaCl_2_/1.2 mM MgSO_4_/1.2 mM KH_2_PO_4_/25 mM NaHCO_3_/11 mM glucose, all reagent were obtained from Sigma) at 37°C gassed. The aortas were sectioned into 2–3 mm transverse segments. Tension was measured using Myograph Pressure System DMT620M (Danish Myo Technology). The aorta rings were placed in the oxygenated organ chambers and equilibrated for 60 min at a constant basal tension of 4 mN. The addition of 1.0 × 10^−6^ M phenylephrine (Phe) established initial vascular activity until maximum contraction. Then, administration of 10^−9^–10^−5^ M acetylcholine (Ach) or sodium nitroprusside (SNP) induced endothelium‐dependent relaxation or endothelium‐independent relaxation. The Ach and SNP induced vasodilatation with cumulative concentrations were recorded.

### 4.15 Cell Culture

HUVECs were isolated from fresh umbilical cord according to our previous reports (Long et al. 2022; Wan et al. 2022). The cells were cultured in endothelial cell medium (Sciencell, 1001) supplemented with 5% fetal bovine serum (Sciencell, 0025), 1% endothelial cell growth supplement (Sciencell, 1052) and 1% penicillin/streptomycin solution (Sciencell, 0503) at 37°C in 5% CO_2_. For the RS model, cells were serially passaged at a 1:2 dilution ratio when reaching 80%–90% confluence. PDL was calculated using the formula: PDL = log_2_(the final cell number harvested/the initial cell number seeded).

Cells were incubated with 5 μM dorsomorphin (MedChemExpress, HY‐13418A) and/or 100 μM citrate (Sigma, 6132‐04‐3) for 2 days to detect protein and MitoSOX, and 6 days to detect SA‐β‐gal.

After cells were incubated with vehicle or 50 μg/mL oligomycin (MedChemExpress, HY‐N6782) for 24 h, culture medium was refreshed and the cells were treated with vehicle or 100 μM citrate respectively and subsequently cultured for 2 days to detect protein and MitoSOX, and 6 days to detect SA‐β‐gal.

### 4.16 Senescence Associated‐β‐Galactosidase (SA‐β‐Gal) Staining

SA‐β‐gal staining was performed as described previously (Debacq‐Chainiaux et al. 2009; Dimri et al. 1995). Cells were washed in PBS, fixed with 2% formaldehyde and 0.2% glutaraldehyde at room temperature for 5 min, and incubated at 37°C overnight with fresh SA‐β‐gal stain solution: 1 mg of 5‐bromo‐4‐chloro‐3‐indolyl β‐d‐galactoside (X‐Gal) per ml (stock = 20 mg of dimethylformamide per ml/40 mM citric acid/sodium phosphate, pH 6.0/5 mM potassium ferrocyanide/5 mM potassium ferricyanide/150 mM NaCl/2 mM MgCl_2_, all reagent were obtained from Sigma).

### 4.17 Immunofluorescence Staining

Cells or tissues were washed in PBS, fixed with 4% paraformaldehyde in PBS at room temperature for 20 min, washed with PBS, permeabilized with 0.5% Triton X‐100 in PBS for 15 min at room temperature. Samples were blocked with blocking buffer (1% BSA and 0.02% Triton X‐100 in PBS) for 1 h at room temperature, followed by incubation at 4°C overnight with primary antibodies p21 (Abcam, ab109520; Proteintech, 10355‐1‐AP), CD31 (R&D technology, AF3628); α‐SMA (Abcam, ab5694); MOMA‐2 (Abcam, ab33451); γ‐H2AX (Cell signaling technology, 2577S); Ki‐67 (Thermo Fisher Scientific, 14‐5698‐82) diluted in blocking buffer and then at room temperature for 1 h with fluorescence‐conjugated secondary antibodies (Jackson, 711‐165‐152; Biolegend, 406402; Invitrogen, A48269; Jackson, 705‐165‐147; Thermo Fisher Scientific, A‐13573; Thermo Fisher Scientific, A48272) diluted in blocking buffer. Three washes in PBS were performed. Nuclei were counterstained with DAPI (Servicebio, G1012). Images were acquired with a microscope and fluorescence intensity values were normalized to nuclear numbers.

### 4.18 Measurement of Oxygen Consumption Rate (OCR)

OCR was measured using an XF24 extracellular flux analyzer (Agilent Technologies) (Plitzko and Loesgen 2018). Cells were cultured on the assay culture plate (Agilent, 102342‐100) for 12 h before the assay (20000 cells per well). Cells were washed with XF cell mitochondrial stress test assay medium (Agilent, 102353‐100) and incubated in a 37°C incubator without CO_2_ for 1 h prior to the assay. In brief, during the measurement, inhibitors of respiratory chain components were serially added to the culture: Oligomycin (1 μM), FCCP (2 μM), and rotenone/antimycin A (0.5 μM). After each addition, the OCR was measured three times (Agilent, 103015‐100).

### 4.19 Reactive Oxygen Species (ROS) Measurement

Cells were incubated with 5 μM of mitochondrial superoxide indicator MitoSOX (Thermo Fisher Scientific, M36008) at 37°C for 10 min, protected from light. Then, wash the cells gently three times with medium. The results were observed under the confocal microscope LSM800 (Zeiss).

### 4.20 Western Blot

Cells were lysed using RIPA lysis buffer, and Western blot was carried out based on published literature (Long et al. 2022). In brief, protein extracts were separated in 8% SDS polyacrylamide gel and transferred onto polyvinylidene fluoride (PVDF) membranes (Thermo Fisher Scientific, 88518). After blocking the membrane for 2 h, the primary antibody was incubated overnight with gentle agitation. The primary antibodies for the target proteins included AMPK (Cell Signaling Technology, 5831S), phosphorylated‐AMPK (p‐AMPK; Cell Signaling Technology, 2531S) and GAPDH (Proteintech, 60004‐1‐1g). Subsequently, the secondary antibody goat anti‐rabbit IgG (Proteintech, SA00001‐2) or goat anti‐mouse IgG (Proteintech, SA00001‐1) was incubated at room temperature for 1 h. The blots were visualized employing a BeyoECL Moon kit (Beyotime, P0018FS). For probing multiple targets with the same membrane, stripping and re‐probing were performed. Briefly, the PVDF membrane was incubated with the stripping buffer containing 62.5 mM Tris–HCl (pH 6.8), 2% SDS, and 100 mM β‐mercaptoethanol for 30 min at room temperature. After washing three times with TBST, the membrane was re‐incubated with primary and secondary antibodies. The protein levels, normalized to GAPDH, were quantified by densitometric analysis using ImageJ.

### 4.21 Statistical Analysis

Statistical analyses were performed utilizing GraphPad Prism software. All data are presented as means ± standard deviation (SD). Two‐tailed student's *t*‐test was used for the comparison between two groups for markers in mice or in HUVECs. One‐way ANOVA was carried out for comparing three or more groups for the senescent marker in HUVECs. Two‐way ANOVA was performed for multiple comparisons with two variables for the determination of citrate concentration in different groups at different times. Log‐rank (Mantel‐Cox) test was used for survival analysis. Significance is indicated as follows: n.s., not significant; *p < 0.05; **p < 0.01; ***p < 0.001 were considered significant.

## Author Contributions

X.‐L.T. and Y.Z. conceived and designed the study. J.‐Y.Q. and F.W. performed the experiments, data processing, and analysis. X.‐T.G., W.‐X.L., J.‐K.L., J.‐Y.D., and X.‐E.L. were involved in discussion and provided academic input. Y.X., J.‐J.D., and A.‐D.W. performed English editing for the manuscript. Y.Z. prepared the manuscript. X.‐L.T. conducted revising and editing. All authors read and approved the final manuscript.

## Disclosure

Permission Statement: All data generated and/or analyzed during this study are permissible.

## Conflicts of Interest

The authors declare no conflicts of interest.

## Supporting information


**Figure S1:** Determination of citrate concentration in aged mice and senescent cells. (a) Serum citrate quantitative data of 2 and 18 months male mice treated vehicle or citrate. (b) Cytosolic citrate quantitative data of young, replicative senescence (RS) and RS treated citrate in HUVECs. Statistical analyses were executed using two‐way ANOVA and one‐way ANOVA. Values are means ± SD. Vehicle and citrate: *n* = 8. *p < 0.05; **p < 0.01; ***p < 0.001.

**Figure S2:** Food and water intake in citrate‐treated mice. (a, b) Average daily food intake (a) and water intake (b) of each mouse in different months of 18 months male mice treated vehicle or citrate. (c) Average daily citrate intake on a per‐body‐weight basis (mg/kg/day) in different months of 18 months male mice treated citrate. Statistical analyses were executed using student's *t*‐test. Values are means ± SD. Vehicle: *n* = 6; Citrate: *n* = 6–8.

**Figure S3:** Effects of citrate on plasma inflammation and lipid fractions. (a–h) High‐sensitivity C‐reactive protein (hs‐CRP) (a), interleukin‐6 (IL‐6) (b), tumor necrosis factor‐α (TNF‐α) (c), matrix metalloproteinase‐9 (MMP‐9) (d), high‐density lipoprotein cholesterol (HDL‐cholesterol) (e), low‐density lipoprotein cholesterol (LDL‐cholesterol) (f), total cholesterol (g), and triglyceride (h) of 18 months male mice after 6‐month vehicle or citrate supplementation. (i–p) hs‐CRP (i), IL‐6 (j), TNF‐α (k), MMP‐9 (l), HDL‐cholesterol (m), LDL‐cholesterol (n), total cholesterol (o), and triglyceride (p) of 8 weeks *ApoE* ^−/−^ male mice after 8‐week high fat diet and vehicle or citrate supplementation. Statistical analyses were executed using student's *t*‐test. Values are means ± SD. Vehicle and citrate: *n* = 8. ***p < 0.001.

**Figure S4:** Citrate reduces γ‐H2AX abundance. (a, b) Immunofluorescence staining of the aorta from vehicle or citrate‐fed mice by antibodies against γ‐H2AX (red). Nuclei (blue) stained by DAPI. Quantitative data for γ‐H2AX positive cells. (c, d) Immunofluorescence staining of senescent HUVECs treated with vehicle or citrate by antibodies against γ‐H2AX (red), and nuclei (blue) stained by DAPI. Quantitative data for γ‐H2AX positive cells. Statistical analyses were executed using student's *t*‐test. Values are means ± SD. Vehicle and citrate: *n* = 8. *p < 0.05; **p < 0.01.

**Figure S5:** Citrate restores the integrity of the damaged intima layer. (a) Immunofluorescence staining of aortic root from vehicle or citrate treatment HFD‐fed *ApoE* ^−/−^ mice by antibodies against CD31 (red). Nuclei (blue) stained by DAPI.

**Figure S6:** Citrate increases Ki‐67 positive smooth muscle cells. (a) Immunofluorescence staining of aortic root from vehicle or citrate treatment HFD‐fed *ApoE* ^−/−^ mice by antibodies against Ki‐67 (yellow) and α‐SMA (red). Nuclei (blue) stained by DAPI. (b) Quantitative data for Ki‐67 positive cells. Statistical analyses were executed using student's *t*‐test. Values are means ± SD. Vehicle and citrate: *n* = 8. **p < 0.01.

**Figure S7:** Effects of citrate at different concentrations on cells. (a) Microscopic imaging of cells treated with 0, 10 μM, 100 μM, 1 mM, 10 mM, and 100 mM of citrate. (b) Quantitative cell counting of cells exposed to 0, 10 μM, 100 μM, 1 mM, 10 mM, and 100 mM of citrate. Statistical analyses were executed using one‐way ANOVA. Values are means ± SD. **p < 0.01.

## Data Availability

All data generated and/or analyzed during this study are included in this article, and the data that support the results of this study are available from the corresponding author upon reasonable request.
