# Supplementary figures and images for: Dietary Citrate Restores Age‐Related Endothelial Cell Mitochondrial Dysfunction and Alleviates Atherosclerosis
Source: Aging Cell. 2025 Sep 4;24(10):e70213. doi: 10.1111/acel.70213 (PMC12507414; doi:10.1111/acel.70213)

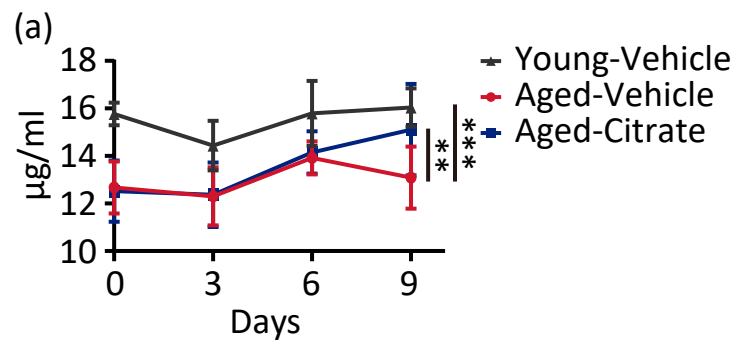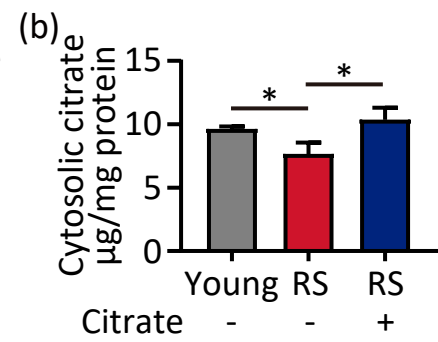

Supplement: Supplementary file 1 — Figure S1: Determination of citrate concentration in aged mice and senescent cells. (a) Serum citrate quantitative data of 2 and 18 months male mice treated vehicle or citrate. (b) Cytosolic citrate quantitative data of young, replicative senescence (RS) and RS treated citrate in HUVECs. Statistical analyses were executed using two‐way ANOVA and one‐way ANOVA. Values are means ± SD. Vehicle and citrate: n = 8. *p < 0.05; **p < 0.01; ***p < 0.001. [file ACEL-24-e70213-s006.pdf]

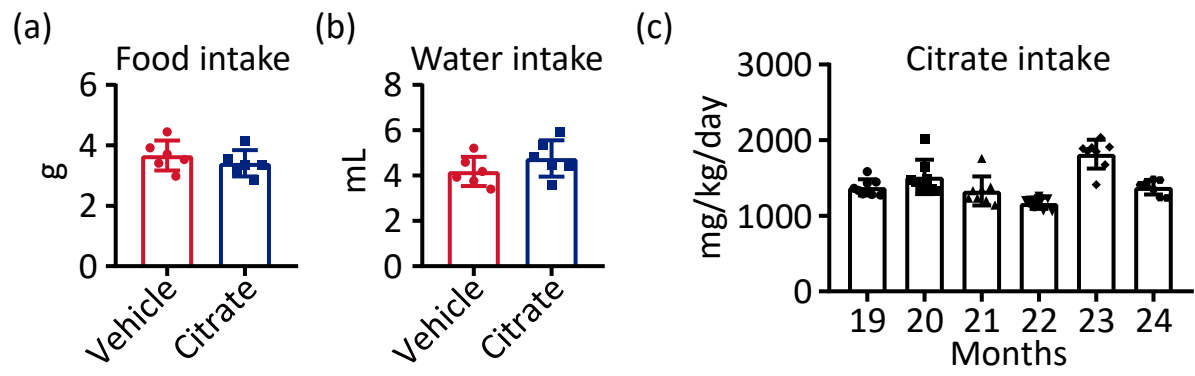

Supplement: Supplementary file 2 — Figure S2: Food and water intake in citrate‐treated mice. (a, b) Average daily food intake (a) and water intake (b) of each mouse in different months of 18 months male mice treated vehicle or citrate. (c) Average daily citrate intake on a per‐body‐weight basis (mg/kg/day) in different months of 18 months male mice treated citrate. Statistical analyses were executed using student's t‐test. Values are means ± SD. Vehicle: n = 6; Citrate: n = 6–8. [file ACEL-24-e70213-s003.pdf]

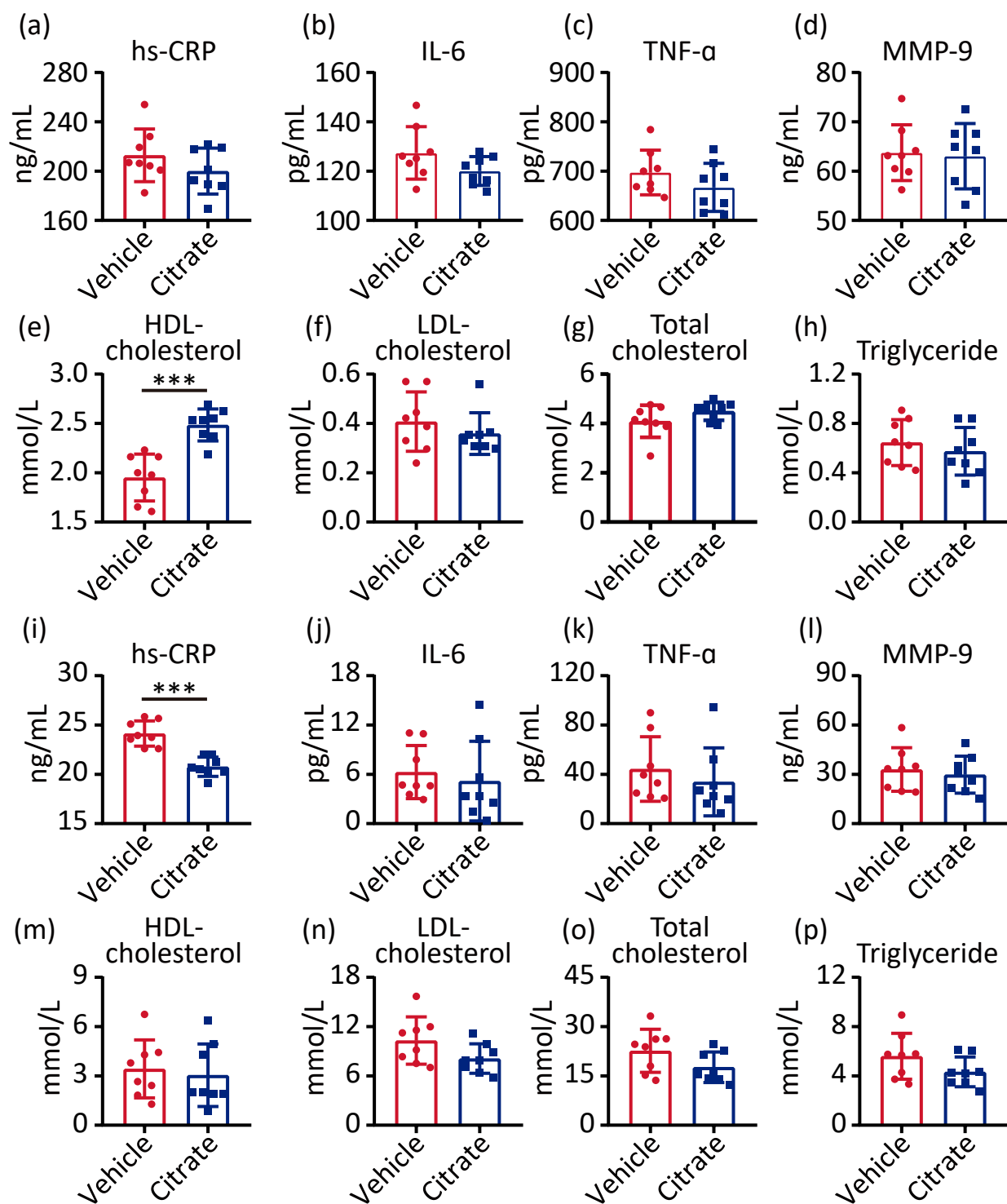

Supplement: Supplementary file 3 — Figure S3: Effects of citrate on plasma inflammation and lipid fractions. (a–h) High‐sensitivity C‐reactive protein (hs‐CRP) (a), interleukin‐6 (IL‐6) (b), tumor necrosis factor‐α (TNF‐α) (c), matrix metalloproteinase‐9 (MMP‐9) (d), high‐density lipoprotein cholesterol (HDL‐cholesterol) (e), low‐density lipoprotein cholesterol (LDL‐cholesterol) (f), total cholesterol (g), and triglyceride (h) of 18 months male mice after 6‐month vehicle or citrate supplementation. (i–p) hs‐CRP (i), IL‐6 (j), TNF‐α (k), MMP‐9 (l), HDL‐cholesterol (m), LDL‐cholesterol (n), total cholesterol (o), and triglyceride (p) of 8 weeks ApoE −/− male mice after 8‐week high fat diet and vehicle or citrate supplementation. Statistical analyses were executed using student's t‐test. Values are means ± SD. Vehicle and citrate: n = 8. ***p < 0.001. [file ACEL-24-e70213-s004.pdf]

(a)

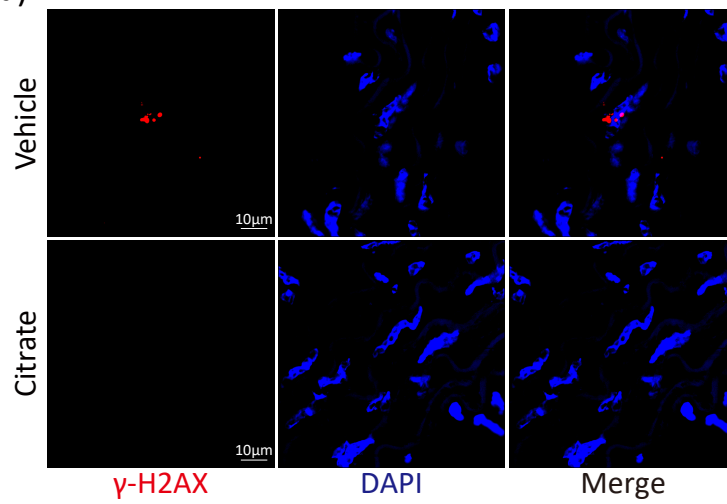

(b)

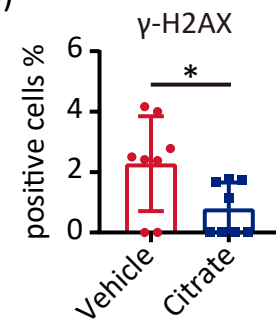

(c)

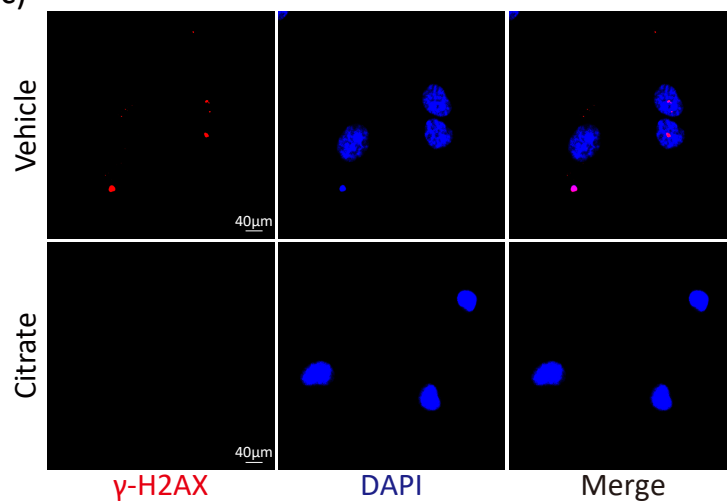

(d)

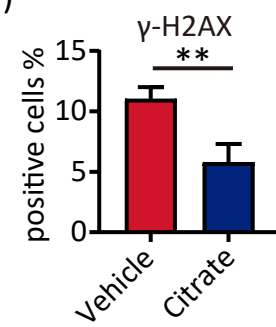

Supplement: Supplementary file 4 — Figure S4: Citrate reduces γ‐H2AX abundance. (a, b) Immunofluorescence staining of the aorta from vehicle or citrate‐fed mice by antibodies against γ‐H2AX (red). Nuclei (blue) stained by DAPI. Quantitative data for γ‐H2AX positive cells. (c, d) Immunofluorescence staining of senescent HUVECs treated with vehicle or citrate by antibodies against γ‐H2AX (red), and nuclei (blue) stained by DAPI. Quantitative data for γ‐H2AX positive cells. Statistical analyses were executed using student's t‐test. Values are means ± SD. Vehicle and citrate: n = 8. *p < 0.05; **p < 0.01. [file ACEL-24-e70213-s005.pdf]

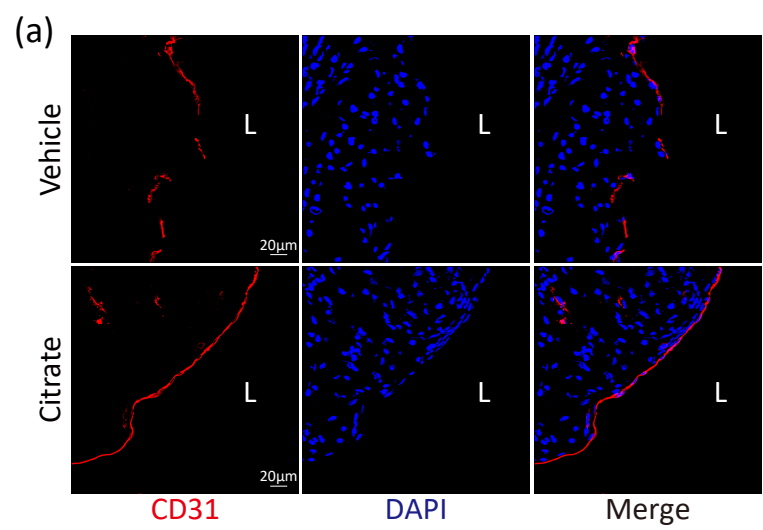

Supplement: Supplementary file 5 — Figure S5: Citrate restores the integrity of the damaged intima layer. (a) Immunofluorescence staining of aortic root from vehicle or citrate treatment HFD‐fed ApoE −/− mice by antibodies against CD31 (red). Nuclei (blue) stained by DAPI. [file ACEL-24-e70213-s001.pdf]

(a)

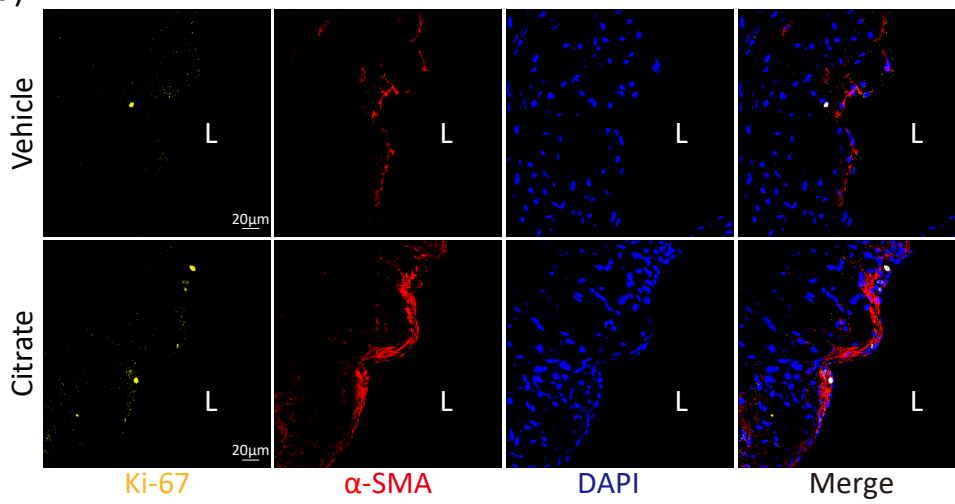

(b)

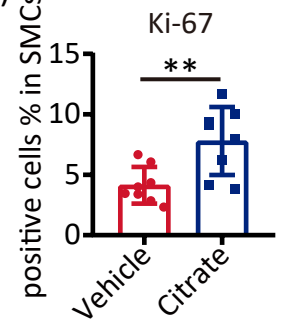

Supplement: Supplementary file 6 — Figure S6: Citrate increases Ki‐67 positive smooth muscle cells. (a) Immunofluorescence staining of aortic root from vehicle or citrate treatment HFD‐fed ApoE −/− mice by antibodies against Ki‐67 (yellow) and α‐SMA (red). Nuclei (blue) stained by DAPI. (b) Quantitative data for Ki‐67 positive cells. Statistical analyses were executed using student's t‐test. Values are means ± SD. Vehicle and citrate: n = 8. **p < 0.01. [file ACEL-24-e70213-s002.pdf]

(a)

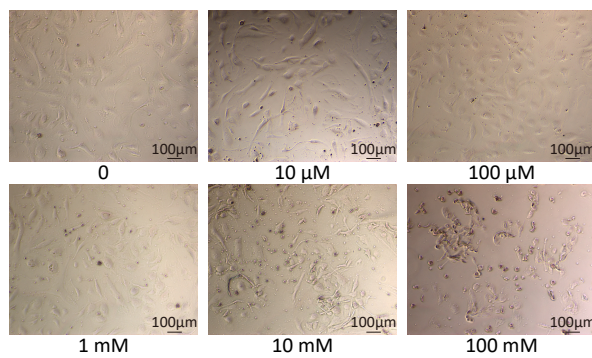

(b)

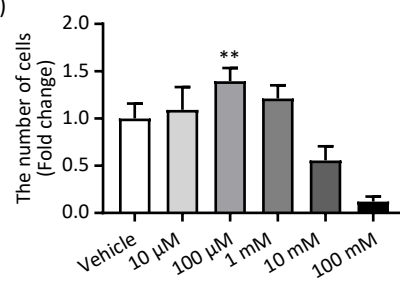

Supplement: Supplementary file 7 — Figure S7: Effects of citrate at different concentrations on cells. (a) Microscopic imaging of cells treated with 0, 10 μM, 100 μM, 1 mM, 10 mM, and 100 mM of citrate. (b) Quantitative cell counting of cells exposed to 0, 10 μM, 100 μM, 1 mM, 10 mM, and 100 mM of citrate. Statistical analyses were executed using one‐way ANOVA. Values are means ± SD. **p < 0.01. [file ACEL-24-e70213-s007.pdf]
